# Emerging Roles of Herpesvirus microRNAs During In Vivo Infection and Pathogenesis

**DOI:** 10.1007/s40139-015-0085-z

**Published:** 2015-07-10

**Authors:** Emily R. Feldman, Scott A. Tibbetts

**Affiliations:** Department of Molecular Genetics & Microbiology, College of Medicine, UF Health Cancer Center, University of Florida, 1200 Newell Dr., R2-291, Gainesville, FL 32610 USA

**Keywords:** Herpesvirus, miRNA, Non-coding RNA, Pathogenesis, Latency, Viral, In vivo

## Abstract

Herpesviridae constitutes a large family of double-stranded DNA viruses that are associated with a wide range of diseases, including herpetic lesions, birth defects, and cancer. Herpesviruses establish lifelong latent infections in part because they are exceptionally adept at modulating the virus/host interface. New insights into the numerous roles of microRNAs (miRNAs) in cell biology, along with the recent appreciation that nearly every host transcript is targeted by at least one miRNA, has fundamentally changed our conceptualization of the virus/host relationship. The identification of miRNAs expressed from nearly all human herpesvirus genomes has led to the speculation that these short non-coding transcripts play essential roles in herpesvirus biology. Because the activity of miRNAs depends upon the transcriptome of the cell in which they are expressed, in vivo systems will be essential for defining the true biological relevance of herpesvirus miRNAs. This review will specifically focus on experimental systems which have investigated the functional role of herpesvirus-encoded miRNAs in viral biology and pathogenesis in vivo.

## Introduction

Over the past decade, the scientific world, including the field of virology, has experienced an exponential growth in the study of non-coding RNA functions. In particular, the plethora of exciting new insights into the numerous roles of microRNAs (miRNAs) in cell biology [1] and the appreciation that nearly every host transcript is targeted by a miRNA [2] has changed the landscape of our conceptualization of the virus/host relationship. Although numerous studies have detected the expression of mature miRNAs from viruses within multiple genera, the early identification of miRNAs in nearly all human herpesvirus genomes [3, 4] has led to the speculation that these molecules play essential roles in herpesvirus biology and pathogenesis.

The Herpesviridae constitutes a large family of enveloped, double-stranded DNA viruses that are associated with a wide range of diseases in humans and animals. Members of this virus family are classified into alpha-, beta-, and gamma-subfamilies based on genomic organization and sequence similarity. Unlike other viruses, following spread to a new host herpesviruses typically undergo an acute lytic phase of replication, and then establish lifelong latency in a distinct cellular compartment. Thus herpesviruses are exceptionally adept at modulating the host/virus interface. Although miRNAs are likely key players in at least some stages of this dynamic, their specific functional roles are currently poorly understood.

miRNAs are small (~22 nucleotide) non-coding RNAs that regulate mRNA translation [1]. Cellular processing of the primary miRNA transcript leads to the release of a stable pre-miRNA hairpin, which is typically further processed by the cellular enzyme Dicer, yielding a miRNA duplex. One strand of the miRNA duplex (the 5′ or the 3′ side of the hairpin, designated as the -5p or -3p strand miRNA accordingly) is then incorporated into the RNA-induced silencing complex (RISC). In the RISC, the miRNA and its target mRNA interact through base pairing that typically requires full complementarity of 6–8 nucleotides called the miRNA seed sequence and thereby, blocking mRNA translation. miRNAs are therefore important regulatory factors whose activity depends upon the transcriptome of the cell in which they are expressed. For this reason, it is likely that the in vivo function of virus-encoded miRNAs may not be easily extrapolated from in vitro studies. Thus, experimental in vivo systems will be essential for defining the true biological relevance of herpesvirus miRNAs. Although the in vivo expression profiles for many viral miRNAs have been carefully elucidated, this review will specifically focus on experimental systems which have investigated the functional role of herpesvirus-encoded miRNAs in the in vivo biology of viral infection.

## Alphaherpesviruses

The alphaherpesvirus subfamily includes the well-known human pathogens herpes simplex virus (HSV)-1 and HSV-2, the causative agents of oral and genital herpetic lesions, and varicella zoster virus (VZV), which causes chickenpox and shingles. The pathogenic outcomes associated with these alphaherpesvirus infections are directly related to the unique ability of these viruses to establish lifelong latency in, and periodically reactivate from, the peripheral nervous system. While no viral miRNAs have thus far been detected during VZV infection, both HSV-1 and -2 encode numerous miRNAs, some of which appear to play key roles in regulating viral infection and pathogenesis. Fortunately, both HSV serotypes are able to establish persistent infections in mice and rabbits, providing robust model systems for studies of viral latency and pathogenesis. Additionally, pseudorabies virus (PRV) represents an important model system that mimics many of the key features of HSV latency establishment in neurons. Because PRV exhibits a broad host tropism, neuronal PRV infections have been studied in a wide range of animals, from mice to chickens to its natural host pigs. Finally, Marek’s disease virus (MDV) is a highly important avian virus that has been classified as alphavirus based on genomic structure and sequence homology. However, MDV exhibits biological properties, including infection of T cells and induction of T cell lymphoma that more closely align with the gammaherpesvirus subfamily and serves as an excellent model system for the study of herpesvirus lymphomagenesis.

### Herpesvirus Simplex Virus-1 and -2

**Fig. 1:**
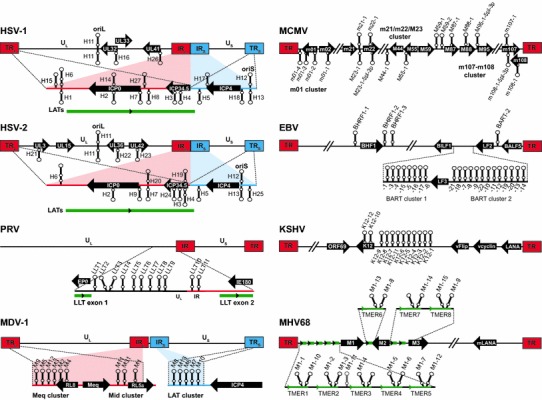
Genomic locations of herpesvirus miRNAs. Genomic maps indicate the number of miRNA stem-loops and approximate locations of miRNAs relative to one another and to nearby transcripts. Pre-miRNA stem-loops depicted above genomes are forward transcribed and those depicted below genomes are reverse transcribed. Nearby coding genes are represented by *black arrows*, and adjacent non-coding transcripts are represented by *green lines*. HSV-1, HSV-2, and MDV-1 encode miRNA precursors within both the internal and terminal repeats, as indicated. Depictions of genomes and transcripts are not to scale (Color figure online)

HSV-1 and -2 establish lifelong latent infections within sensory neurons of the trigeminal ganglia (TG) and sacral dorsal root ganglia (DRG), respectively. HSV-1 reactivation events induce a range of diseases from common oral cold sores to herpes stromal keratitis to rare cases of encephalitis, whereas HSV-2 reactivation from the DRG typically results in genital herpetic lesions. HSV-1 and -2 encode 20 and 17 potential pre-miRNA stem-loops [5–9], respectively, many of which are distributed in clusters within and flanking the latency-associated transcript (LAT) (Fig. 1). Although several pre-miRNAs display positional homology between HSV-1 and HSV-2, with the exception of three molecules that retain identical seed sequences (hsv-miR-H2-3p, -H7-5p, -H11-3p), the mature HSV-1 and -2 miRNAs display little sequence similarity [8].

Notably, initial work on the HSV-1 LAT-encoded miRNAs suggested that although hsv1-miR-H2, -H4, -H5, and -H6 were detectable in TGs during latency, these molecules may be dispensable for latent infection: in wild-type mice, LAT promoter mutants that lacked expression of these miRNAs replicated normally and established wild-type levels of latent infection [10]. However, further work examining the function of individual molecules has led to the conclusion that single miRNAs may play important roles in infection which are obscured by combinatorial miRNA mutations.

One HSV-1 miRNA of great interest is hsv1-miR-H2, which lies antisense to and regulates translation of the key immediate-early gene ICP0 (Fig. 1) [9], and displays a seed sequence identical to its HSV-2 positional miRNA homolog. To examine the importance of hsv1-miR-H2 for in vivo infection, Jiang et al. constructed a precise mutant on the highly virulent wild-type McKrae virus background which disrupts the pre-miRNA structure, but retains the amino acid sequence of ICP0, an E3 ubiquitin ligase that is known to facilitate lytic replication and reactivation from latency [11•]. Ocular infection of Swiss Webster mice with the H2 mutant virus led to the increased penetrance of herpes encephalitis as compared to the parental strain (survival 86 vs 52 %); however, this was not due to enhanced replication at the site of infection, as ocular titers were equivalent between wild-type and mutant virus. In contrast, whereas the number of latent viral genomes in the TG were not statistically different at 30 days post-inoculation (dpi) between the two viruses, TG explants from H2 mutant-infected mice demonstrated an increased rate of reactivation from latency [11•], offering a potential explanation for the observed increase in neurovirulence. Consistent with this, mutation of neuron-specific miR-138, which regulates ICP0 transcripts, results in increased neurovirulence in vivo [12].

Of additional interest for both HSV-1 and HSV-2 are the positional miRNA homologs at the miR-H6 position, which lie just outside of the crucial LAT promoter (Fig. 1), and are regulated by LAT promoter activity [10, 13]. Moreover, hsv1-miR-H6 regulates the essential immediate-early gene ICP4 [9]. Although hsv2-miR-H6 does not directly regulate ICP4 [9, 14], ICP4 suppresses the expression of other key LAT-encoded HSV-2 miRNAs [13], suggesting that intricate regulation of the bidirectional LAT promoter may play a critical role in regulation of the balance between latency and reactivation. To determine whether hsv2-miR-H6 played a key role in in vivo infection, Tang et al. disrupted miR-H6 expression by inserting an upstream poly(A) sequence, which abrogated miR-H6 expression but did not affect levels of HSV-2 LAT or viral DNA copy number [14]. Following the inoculation of mice (intraocular) or guinea pigs (intravaginal), the polyA miR-H6 mutant replicated and established latency in ganglia (TG for mice, DRG for guinea pigs) at levels comparable to wild-type virus. Importantly though, guinea pigs infected with the mutant displayed complete attenuation of neurological symptoms (0 % for the mutant, compared to 37.5 and 62.5 % for revertant and wild-type HSV-2, respectively), demonstrating a key role for hsv2-miR-H6 in HSV-2 pathogenesis through unknown means likely involving regulation of multiple host and viral components.

### Pseudorabies Virus

Pseudorabies virus (PRV, SuHV-1) is a highly contagious alphaherpesvirus that primarily infects swine, but also infects a wide range of domestic livestock and forest-dwelling mammals [15]. Although PRV infection is frequently asymptomatic, infection of young animals can result in severe neurological, respiratory, and reproductive system manifestations which are characteristic of Aujeszky’s disease (AD). Depending upon virus strain, PRV infection can result in nearly 100 % fatality of piglets [15].

Following intranasal inoculation, PRV establishes latency in the neurons of TG. During in vivo latency, the LAT locus is the only region of the PRV genome that is transcriptionally active. The largest transcript within the LAT locus is the full-length, 8.4 kb polyadenylated large latency transcript (LLT), which is expressed at high levels during latency and is spliced to yield a 4.6 kb stable intron [16]. Recent work has demonstrated that the PRV LLT functions in part as a primary miRNA precursor which encodes 11 pre-miRNA stem-loops, all located within the LLT intron (Fig. 1) [7, 17, 18]. To determine the function of PRV miRNAs during in vivo infection, Mahjoub, et al. generated a cluster deletion mutant that lacked 9 of the 11 PRV pre-miRNA stem-loops [19•].

Following i.n. infection of German Landrance pigs, the mutant virus replicated in nasal swabs to levels slightly higher than wild-type virus, demonstrating that these PRV miRNAs are dispensable for acute replication. Similarly, despite detecting expression of the miRNAs from the WT virus genome in TG, deletion of the 9-cluster miRNAs had little effect on latency establishment, as determined by PCR for viral genome at 62 dpi. Although these results did not reveal a crucial role for these PRV miRNAs during in vivo infection, the effects of the remaining PRV miRNAs in pathogenesis remain to be determined.

### Marek’s Disease Virus

Marek’s disease virus-1 (MDV-1, GaHV-2) is a highly contagious avian alphaherpesvirus that is the causative agent of the T-cell lymphoma for which it is named. Today, MDV-1 infection of chickens represents an excellent model system for studying virus-induced neoplastic diseases [20, 21]. Although there are several circulating MDV-1 strains, it is important to note that individual strains vary in virulence, and have thus been classified as mild (m), virulent (v), very virulent (vv), and very virulent plus (vv+) [22]. While the penetrance of disease and the time of onset vary with strain virulence, MDV-1 infection reproducibly results in T-cell lymphoma in a wide range of chicken lines. As a result, MDV-1 lymphomagenesis has offered perhaps the most direct and important means by which to define a central role for virus-encoded miRNAs in tumorigenesis.

MDV-1 encodes a total of 14 pre-miRNAs, each of which are duplicated within their respective terminal and internal repeat regions (Fig. 1) [7, 23, 24]. As for other herpesviruses, the MDV-1 miRNAs are clustered, with two clusters surrounding the important *meq* oncogene and another cluster incorporated within the LAT. Interestingly, several MDV-1 miRNAs are much more highly expressed in tumor tissue than non-tumor tissue, suggesting the possibility that the miRNAs may contribute to lymphomagenesis [25]. Moreover, only miRNAs from the Meq cluster are differentially expressed by the very virulent strains of MDV-1, leading to the speculation that these miRNAs play a greater role in disease progression [26].

To define the contribution of MDV-1 miRNAs to neoplastic growth in vivo, multiple research groups have generated specific miRNA mutants on parental MDV-1 strains of varying virulence. In a seminal manuscript for the viral miRNA community, Zhao, et al. compared tumorigenesis of the virulent pRB-1B5 (a BAC-containing version of RB-1B) strain to a mutant of the parental strain lacking expression of the cluster of six miRNAs lying upstream of Meq [27]. While wild-type virus was lethal in 100 % of animals within 7–8 weeks post-inoculation, pRB-1B5 lacking expression of these six miRNAs was completely attenuated. Surprisingly, a two nucleotide seed sequence mutant of the single miRNA mdv1-miR-M4, a functional ortholog of the important cellular oncomir miR-155 [28], was equally attenuated for lethality, resulting in 100 % survival of infected animals. Thus, these data demonstrated an essential role for mdv1-miR-M4 in MDV-1 lymphomagenesis, and further substantiated the conclusion that miR-155 dysregulation can be a principal factor in the development of lymphoid tumors.

It is notable that a mdv1-miR-M4 mutant on the background of the very virulent parental strain GX0101 demonstrated a slower onset of malignant disease, and reduced but did not abolish disease penetrance (100 % wild-type, 18 % miR-M4 mutant), indicating that M4 is not absolutely essential for oncogenesis [29•]. Although this study utilized a higher inoculation dose and increased experimental time frame than the previous experiment, these findings may indicate that additional viral factors may also contribute to the pathogenesis of MDV-1 strains with enhanced virulence. In support of this, Teng et al. used additional single miRNA mutants to demonstrate divergent roles for other Meq-clustered miRNAs in the induction of MD lymphoma [30•]. While 100 % of parental GX0101-infected birds succumbed to infection by 75 dpi, individual mutants of mdv-1-miR-M2, -M3, -M5, -M9, or -M1 displayed an incidence of disease ranging from 14 to 48 %, demonstrating a contribution of these additional miRNAs to tumorigenesis. While the differences between these studies must be carefully delineated in future work, these findings clearly demonstrate an essential role for the Meq-clustered miRNAs in the genesis of MDV-1-associated tumors.

## Betaherpesviruses

The most prominent member of the betaherpesvirus subfamily is human cytomegalovirus (HCMV). HCMV is a ubiquitous virus that is primarily asymptomatic in immunocompetent humans, but is a very significant cause of morbidity and mortality in patients with compromised immunity, and is the leading cause of birth defects in industrialized countries. Cytomegalovirus infections characteristically induce a lytic infection in glandular epithelial cells, particularly within the salivary gland, and establish persistent infections that likely involve cycles of latency, reactivation, and lytic infection. Betaherpesviruses such as HCMV, and human herpesviruses-6A, -6B, and -7, are highly species specific. Thus the development of experimental animal models for the human viruses has not been successful. Although the gene structure of murine cytomegalovirus (MCMV) differs significantly from that of HCMV, MCMV exhibits important biological properties in mice that overlap with that of HCMV in humans. Thus MCMV infection of mice has served as an outstanding model for understanding in vivo aspects of cytomegalovirus replication and pathogenesis, and has been a particularly valuable tool for defining the function of virus-encoded genes in evasion of the immune response.

### Murine Cytomegalovirus

HCMV encodes 11 pre-miRNA stem-loops yielding 17 detectable mature miRNAs, and MCMV encodes 18 pre-miRNA stem-loops yielding up to 29 detectable mature miRNAs [7, 31]; however, the HCMV and MCMV miRNAs exhibit little or no homology [32]. Nevertheless, as has been the case for protein-coding genes, the study of MCMV miRNAs is likely to elucidate valuable insights into the complex relationship between betaherpesviruses and their natural hosts.

MCMV miRNAs are arranged into three distinct clusters which have been annotated as the m01 cluster, the m21/m22/m23 cluster, and the m107–m108 cluster (Fig. 1). To date, only the m21/m22/M23 cluster miRNAs have been carefully characterized in vivo. Dolken et al. generated a virus with 17 point mutations in the pre-miRNA stem-loop of two of the most highly expressed MCMV miRNAs, mcmv-miR-M23-2 and mcmv-miR-m21-1 [33]. To determine the biological relevance of these miRNAs in the context of in vivo infection, the authors intravenously infected both B6 and BALB/c mice, and measured viral titers in salivary glands and lungs at 14 dpi. The salivary glands are the primary site of MCMV replication and shedding, and are an immune-privileged site from CD8 T cells, but not CD4 T cells and NK cells [34]. Although both BALB/c and B6 mice are susceptible to MCMV infection, B6 mice much more efficiently control salivary gland replication due to the presence of the activating NK cell receptor Ly49H. Interestingly, salivary gland replication of the miR-M23/m21 mutant virus was attenuated nearly 100-fold in B6 mice, but was reduced only twofold in BALB/c mice, suggesting that NK cell-mediated control is compromised in the absence of miR-M23/m21 expression [33]. Consistent with this conclusion, replication of the mutant virus in B6 mice was restored to wild-type levels upon NK cell depletion. Thus this work demonstrated a key role for a MCMV miRNA in evasion of the immune response, and was the first to demonstrate the in vivo importance of a herpesvirus miRNA.

## Gammaherpesviruses

The gammaherpesvirus subfamily includes the important human pathogens Epstein–Barr virus (EBV) and Kaposi’s sarcoma (KS)-associated herpesvirus (KSHV, HHV-8). EBV is a highly ubiquitous human pathogen, chronically infecting greater than 90 % of the adult human population worldwide. Moreover, EBV infection is associated with the development of numerous types of malignancies, including Burkitt’s B-cell lymphoma, nasopharyngeal carcinoma (NPC), and Hodgkin’s lymphoma. KSHV is a lymphotropic gammaherpesvirus that infects endothelial cells and establishes latency in mature circulating B cells. KSHV infection is associated with a variety of malignancies including KS, primary effusion lymphoma (PEL), and multicentric Castleman’s disease (MCD).

Although an extensive amount of work has been directed toward defining the function of individual protein-coding genes and non-coding RNAs during EBV and KSHV infection in vitro, determining the specific roles that these elements play in vivo is an enormous obstacle due to their strict species specificity. Thus, an important advance has come from the recent use of humanized mice, which utilizes transplants of human hematopoietic stems cells (HSCs) to partially reconstitute the immune systems of severely immunocompromised mice [35]. However, humanized mouse systems are extremely expensive, do not support long-term infections, and by necessity employ highly immunodeficient mouse strains; thus such models are not optimal for understanding all aspects of virus/host relationship. Nevertheless, humanized mouse systems provide an opportunity to examine some in vivo functions of human viral genes in the context of its parental virus. Similarly, ectopic expression of viral genes or gene clusters in adoptive immune reconstitution and transgenic mouse studies have proved to be of successful means to define functions of viral genes independent of virus infection.

Like EBV and KSHV, murine gammaherpesvirus 68 (MHV68, γHV68, MuHV-4) establishes latent infection in B cells and results in the induction of lymphoproliferative disease and B-cell lymphoma in the setting of a compromised immune system. Thus MHV68 infection of mice has proven to be a facile and robust system for examining cellular and molecular aspects of the complex relationship between a gammaherpesvirus and its natural host.

### Epstein–Barr Virus

Following transfer of virus to a naive host, EBV undergoes lytic replication in the oral epithelium and establishes lifelong latency in circulating memory B cells. Although EBV infection is typically asymptomatic, acute or chronic infection in the setting of immunocompromise or alongside essential cofactors can lead to the development of multiple types of epithelial or lymphoid malignancies. Thus understanding the function of EBV miRNAs in the context of B cell and epithelial biology is of utmost interest. The 172 kb EBV genome encodes 25 pre-miRNA stem-loops that yield up to 44 mature miRNAs [7, 36]. The EBV miRNAs are located in three distinct clusters, with one cluster flanking the the Bcl-2 homolog BHRF1, and the others located in two separate clusters within the BART RNA transcript (Fig. 1).

To determine the in vivo function of the three BHRF1 cluster miRNAs, Wahl et al. generated a virus carrying deletions of ebv-miR-BHRF1-1, ebv-miR-BHRF1-2, and ebv-miR-BHRF1-3 [37•]. Following the direct injection of virus into the spleens of humanized mice, the authors observed a significant delay in the accumulation of the mutant virus DNA in the peripheral blood as compared to that of wild-type virus. However, this delay did not affect overall pathogenesis, as the mutant virus resulted in a similar penetrance of EBV+ tumors. Thus, these results indicated that the BHRF1-cluster miRNAs may facilitate initial acute replication and systemic spread of EBV, but that they do not play an essential role in EBV oncogenesis. It is noteworthy that these in vivo findings contrast with the established role of the BHRF1 miRNAs in potentiation of B-cell transformation in vitro [38], which may suggest that other virus or host factors perform activities redundant to the BHRF1 miRNAs in an in vivo system.

Further work using an irradiated mouse model system examined the role of the BART cluster 2 miRNA ebv-miR-BART7-3p in promoting NPC metastases. ebv-miR-BART7-3p is highly expressed in NPC samples [39••] and positively correlates with tumor metastasis and clinical stage. To determine whether ebv-miR-BART7-3p facilitates metastatic transition in vivo, Cai et al. generated EBV-negative NPC cells lines stably expressing ebv-miR-BART7-3p. Cells were then transplanted under the liver capsule of irradiated mice, and lymph node metastases were observed after 3 weeks. Consistent with in vitro findings, lymph node metastases were observed in 83 % of mice transplanted with NPC cells expressing ebv-miR-BART7-3p, compared to only 17 % of mice transplanted with control NPC cells, suggesting that ebv-miR-BART7-3p stimulates epithelial-to-mesenchymal transition (EMT). The authors further demonstrated that this transition was mediated at least in part through miRNA targeting of the PI3K/Akt/GSK-3β pathway and subsequent downregulation of PTEN [39••].

### Kaposi’s Sarcoma-Associated Herpesvirus

KSHV encodes 12 pre-miRNAs which yield 25 detectable mature miRNAs (Fig. 1), all located within the latency-associated region of the genome (10 in the intronic region between the protein-coding genes K12 and v-FLIP, and 2 within the K12 ORF sequence). Due to the prominent role of host miR-155 in B-cell differentiation and cancer [40], the miR-155 ortholog kshv-miR-K12-11 has been of tremendous interest. To determine whether kshv-miR-K12-11 functions similar to miR-155 to facilitate B-cell development, Boss et al., transplanted human hematopoietic progenitors stably expressing miR-K12-11 or miR-155 into NOD/LtSz-scid IL2Rγ^null^ mice and monitored immune reconstitution over time [41]. Importantly, ectopic expression of either kshv-miR-K12-11 or miR-155 guided a significant expansion of human CD19+ splenic B cells compared with control mice. Validating these findings, stable expression of kshv-miR-K12-11 in HSCs resulted in mature B-cell expansion in the periphery and an increase in the number of developing pre-B cells in the bone marrow [42•]. Moreover, the latency locus of KSHV complemented the B-cell development and response phenotype of miR-155-deficient mice, demonstrating that miR-K12-11 indeed can mimic miR-155 functions in vivo [43•, 44, 45]. Together, these studies demonstrated a highly important functional role for kshv-miR-K12-11 in modulating B-cell differentiation and provided great insight into the potential for a single virus-encoded miRNA to potently alter the fate of infected cells in vivo.

### Murine Gammaherpesvirus 68

Murine gammaherpesvirus 68 (MHV68, γHV68, MuHV-4) is a natural pathogen of wild rodents, including mice, and MHV68 infection of laboratory mice has served as an important model for the in vivo study of gammaherpesvirus latency and pathogenesis. MHV68 encodes 14 pre-miRNA stem-loops which yield up to 28 mature miRNAs [7, 46–48]. Interestingly, the 14 stem-loop sequences are incorporated into 8 primary pol III transcripts (Fig. 1) that carry a viral tRNA (vtRNA)-like element at the 5′ end. These tRNA-miRNA-encoding RNAs (TMERs), which are approximately 250 bp in length, are processed in a Drosha-independent manner prior to entering the canonical miRNA processing pathway. Although it has been long-known that the TMER transcripts are highly expressed in latently infected cells and in virus-associated malignancies [49, 50], a functional role for these miRNAs during infection has only recently been uncovered.

Using differing approaches to generate combinatorial miRNA mutant viruses, two groups, including our own, have now demonstrated roles for the MHV68 miRNAs in both latency and pathogenesis. Following intranasal inoculation of B6 mice with MHV68.Zt6, a recombinant MHV68 with deletions of pre-miRNA stem-loops encoded by TMERs 1–5, 7, and 8, and a transcriptional stop upstream of the TMER6 miRNAs, underwent normal acute replication in lungs in vivo, demonstrating that the MVH68 miRNAs are dispensable for lytic replication [51••]. Nevertheless, this mutant displayed a 2.3-fold reduction in latency establishment and reduced numbers of infected memory B cells, suggesting that the virus-encoded miRNAs may facilitate differentiation of infected B cells during latency establishment. Similarly, Diebel et al. generated a combinatorial TMER mutant virus, MHV68.TKO, which carries deletions in the pol III promoters of each of the TMERs, and found that TMER expression was dispensable for acute replication [52••]. In contrast to the latency attenuation observed following infection with MHV68.Zt6, the MHV68.TKO mutant displayed slightly elevated frequencies of infected cells at 14 dpi. However, the discrepancies between these two mutants may be due to the lack of vtRNA expression by the promoter mutant virus: while MHV68.Zt6 expresses normal levels of the TMER-encoded vtRNAs, MHV68.TKO lacks expression of the entire TMER transcript including vtRNAs. These findings may suggest that the virus-encoded vtRNAs themselves play a functional role in vivo.

Strikingly, despite these subtle latency phenotypes in wild-type animals, the combinatorial miRNA mutant viruses displayed an impressive attenuation of lethal pneumonia in BALB/c mice deficient in IFNγ. While only 20 % of mice infected with wild-type virus survive the pulmonary inflammatory disease observed in these mice, 100 % of mice inoculated with MHV68.Zt6 survive infection [51••]. Similarly, 80 % of mice infected with the MHV68.TKO virus survive infection [52••]. Although the specific targets of the MHV68 miRNAs are yet to be resolved, together these reports demonstrate an essential role for MHV68-encoded miRNAs in this robust model of viral disease. Further, these findings also suggest that while herpesvirus-encoded miRNAs may only subtly regulate chronic infection, their presence may strongly influence the development or outcome of disease.

## Conclusions

Despite the rapid growth of miRNA research in recent years, the understanding of the specific functions of virus-encoded miRNAs during infection is still in its infancy. In particular, due to their likely subtle and redundant effects, defining the biological relevance of virus-encoded miRNAs in in vivo systems poses a substantial hurdle. Yet, important advances in the field have been made in the last few years, and new themes appear to be emerging.

### Herpesvirus miRNAs are Largely Dispensable for Acute Replication

Experiments in the HSV-1, HSV-2, PRV, and MHV68 systems have clearly demonstrated that individual or combinatorial miRNA mutant viruses are able to replicate normally in vivo, a finding which is generally consistent with parallel in vitro studies. Although an MCMV miRNA mutant exhibited severely reduced titers in vivo, this observation was definitively linked to evasion of NK cell evasion, providing an elegant demonstration of the potential of a single viral miRNA to manipulate host immunity.

### Herpesvirus miRNAs Significantly Contribute to Pathogenesis

Importantly, despite the lack of obvious role for herpesvirus miRNAs in acute infection, and the apparently subtle roles of herpesvirus miRNAs in latency and reactivation, experiments in multiple in vivo systems have now clearly demonstrated an essential role for viral miRNAs in pathogenic outcomes. For example, within their respective systems, miRNA mutants are severely attenuated for HSV-1 neurovirulence, MHV68 inflammatory pneumonia, and MDV-1 lymphomagenesis. Moreover, studies with KSHV K12-11 and EBV BART-7-3p validate the concept that individual viral miRNAs can very significantly impact the replication and metastatic potential of infected cells. Although this theme does not apparently extend to the EBV BHRF1-cluster miRNAs, other EBV miRNAs may contribute to oncogenesis, and the EBV BART cluster 1 and 2 miRNAs are yet to be carefully tested in this system.

Based on the likelihood that viral miRNAs function in a cell-type specific manner, and owing to the requirement of target sequence specificity, it would seem that defining the specific functions of these molecules in highly complex in vivo systems will surely prove to be extremely difficult. Nevertheless, the recent advances described here have yielded critical new insight into the potent actions of some viral miRNAs, and provide excitement for the future of in vivo herpesvirus non-coding RNA experimentation.
